# Hepatic Hilar Nerve Block as an Adjunct to Moderate Sedation for Microwave Ablation of Hepatocellular Carcinoma: A Case Report

**DOI:** 10.7759/cureus.102710

**Published:** 2026-01-31

**Authors:** Renato Abu Hana, Ruben G Ortiz Cordero, Vedant Garg, Grit A Adler, Vinicius Adami Vayego Fornazari

**Affiliations:** 1 Division of Vascular and Interventional Radiology, Department of Radiology, University of Florida College of Medicine - Jacksonville, Jacksonville, USA; 2 Department of Radiology, University of Florida College of Medicine - Jacksonville, Jacksonville, USA

**Keywords:** hepatic hilar nerve block, hepatocellular carcinoma, interventional radiology, microwave ablation, regional anesthesia

## Abstract

Effective pain control is a key determinant of safety and technical success during percutaneous liver tumor ablation, particularly for microwave ablation (MWA), which is associated with significant visceral pain. General anesthesia (GA) is frequently used but may be unavailable in many institutions and may increase peri-procedural risk in patients with cirrhosis. We report a technically focused case highlighting the educational value of a hepatic hilar nerve block as an adjunct to moderate sedation during MWA of hepatocellular carcinoma (HCC). A 71-year-old man with hepatitis C-related cirrhosis and residual HCC after transarterial chemoembolization underwent percutaneous MWA using moderate sedation combined with a hepatic hilar nerve block. The procedure was completed successfully without escalation to GA, with excellent intra- and post-procedural analgesia and no complications. During hospitalization, the patient reported only mild pain with a visual analog scale (VAS) score of 3/10, which was controlled with acetaminophen alone without opioids. At one-week follow-up, the patient reported no pain with a VAS score of 0/10.

## Introduction

Hepatocellular carcinoma (HCC) is the most common primary liver malignancy and most frequently arises in the setting of cirrhosis, often related to chronic viral hepatitis, alcohol-associated liver disease, or nonalcoholic steatohepatitis [1,2]. Underlying hepatic dysfunction plays a central role in treatment selection and clinical outcomes, highlighting the importance of individualized and multidisciplinary management [1-3].

Therapeutic options for HCC encompass a broad range of modalities, including systemic therapy, surgical resection or transplantation, catheter-directed intra-arterial treatments, and percutaneous image-guided thermal ablation [1-3]. In select patients with early-stage disease, percutaneous thermal ablation offers a minimally invasive, curative-intent option with favorable oncologic outcomes and low morbidity, establishing it as a cornerstone therapy among interventional radiology treatment options [1,2,4]. Microwave ablation (MWA) enables the creation of larger and more predictable ablation zones; however, it is frequently associated with significant visceral pain due to hepatic capsular and parenchymal heating [4,5]. Consequently, many centers perform MWA under deep sedation or general anesthesia, which may increase peri-procedural risk, particularly in patients with advanced liver disease [6,7].

Targeted regional anesthesia techniques, such as hepatic hilar nerve block, provide an effective alternative by improving procedural analgesia while mitigating the risks associated with general anesthesia [5,8,9]. The hepatic hilar nerve block is a regional technique targeting the nerves of the hepatic plexus at the hepatic hilum, where they run alongside the portal vein and hepatic artery within the Glisson sheath [4,5]. Current literature describes the hilar nerve block as a safe and effective adjunctive analgesic strategy [5]. Additionally, it has been reported to decrease analgesia requirements and to lower fentanyl and midazolam use without compromising procedure success or increasing adverse events [5]. The hepatic hilar nerve block can target visceral pain pathways originating from the liver parenchyma, making it preferred over the superficial techniques (e.g., transversus abdominis plane (TAP) block) for interventional procedures, such as percutaneous liver ablation [9-11]. This case report discusses the successful use of a CT-guided hepatic hilar nerve block, highlighting its benefits as an effective, targeted regional analgesia strategy for patients undergoing ablation in whom general anesthesia or deep sedation is undesirable due to high surgical risk.

## Case presentation

**Figure 1:**
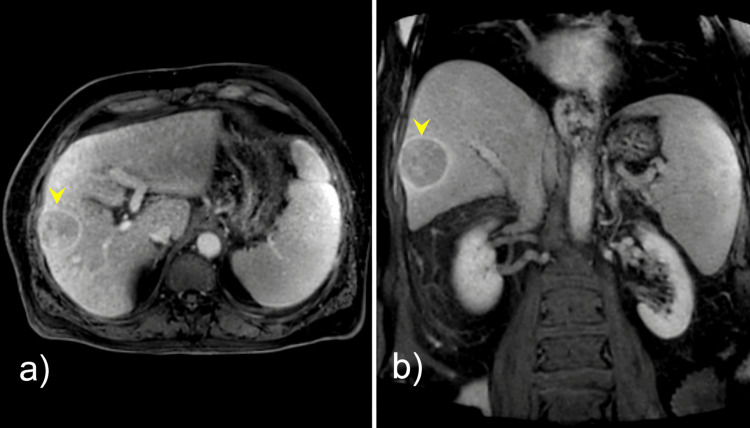
Contrast-enhanced computed tomography (CT) images of the abdomen (A) Axial and (B) coronal contrast-enhanced computed tomography (CT) images of the abdomen demonstrating cirrhotic liver morphology with a lesion (yellow arrowheads) in hepatic segment V with arterial phase hyperenhancement and washout on delayed phases, consistent with a Liver Imaging Reporting and Data System (LI-RADS) 5 lesion.

**Figure 2:**
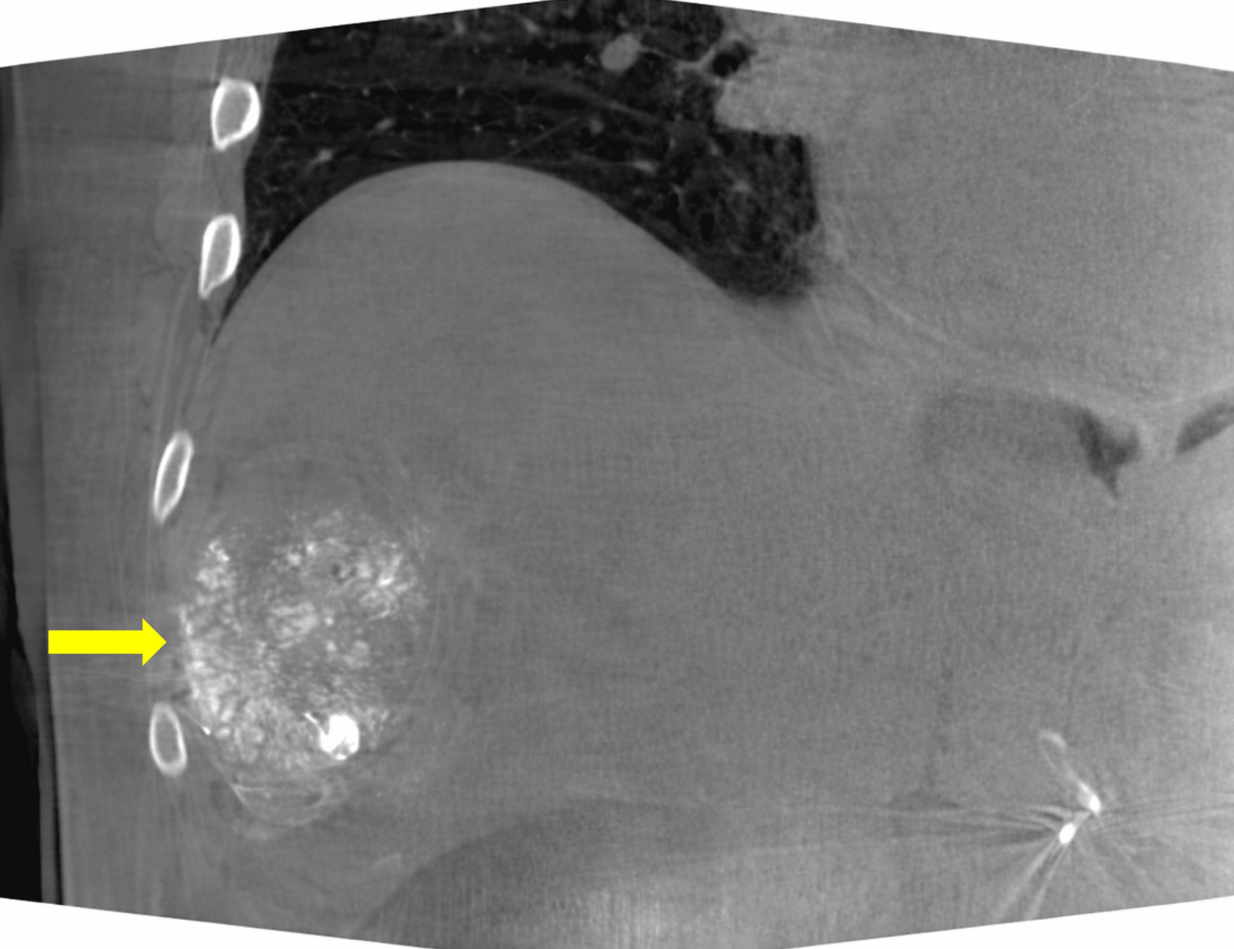
Coronal contrast-enhanced computed tomography (CT) image demonstrating a residual lesion (yellow arrow) in hepatic segment V containing radiopaque material (lipiodol) after transarterial chemoembolization

A 71-year-old man with hepatitis C-related cirrhosis was diagnosed with a 5.0-cm Liver Imaging Reporting and Data System (LI-RADS) 5 lesion in hepatic segment V, consistent with HCC (Figure 1), in accordance with current imaging diagnostic criteria [2]. Due to comorbidities, portal hypertension, and tumor characteristics, the patient was not a candidate for surgical resection or liver transplantation. After initial transarterial chemoembolization, follow-up imaging demonstrated residual arterial enhancement suggestive of viable tumor (Figure 2).

Prior to the procedure, the patient reported intermittent fatigue. Laboratory evaluation showed hemoglobin and hematocrit within normal limits, with leukopenia (3.59 × 10³/µL) and thrombocytopenia (51 × 10³/µL). The comprehensive metabolic panel demonstrated hypoalbuminemia (3.1 g/dL), elevated total bilirubin (2.6 mg/dL), and elevated aspartate aminotransferase (AST; 81 IU/L). The international normalized ratio (INR) was mildly elevated at 1.2. Hepatic function was classified as Child-Pugh class B (score of 7) with a Model for End-Stage Liver Disease-Sodium (MELD-Na) score of 13, and the patient was classified as American Society of Anesthesiologists (ASA) physical status class III. These findings were not considered contraindications to proceeding with ablation of the residual tumor.

Given the patient's comorbidities, the procedure was planned to be performed under moderate sedation with adjunctive hepatic hilar nerve block rather than general anesthesia.

Hepatic hilar nerve block technique

**Figure 3:**
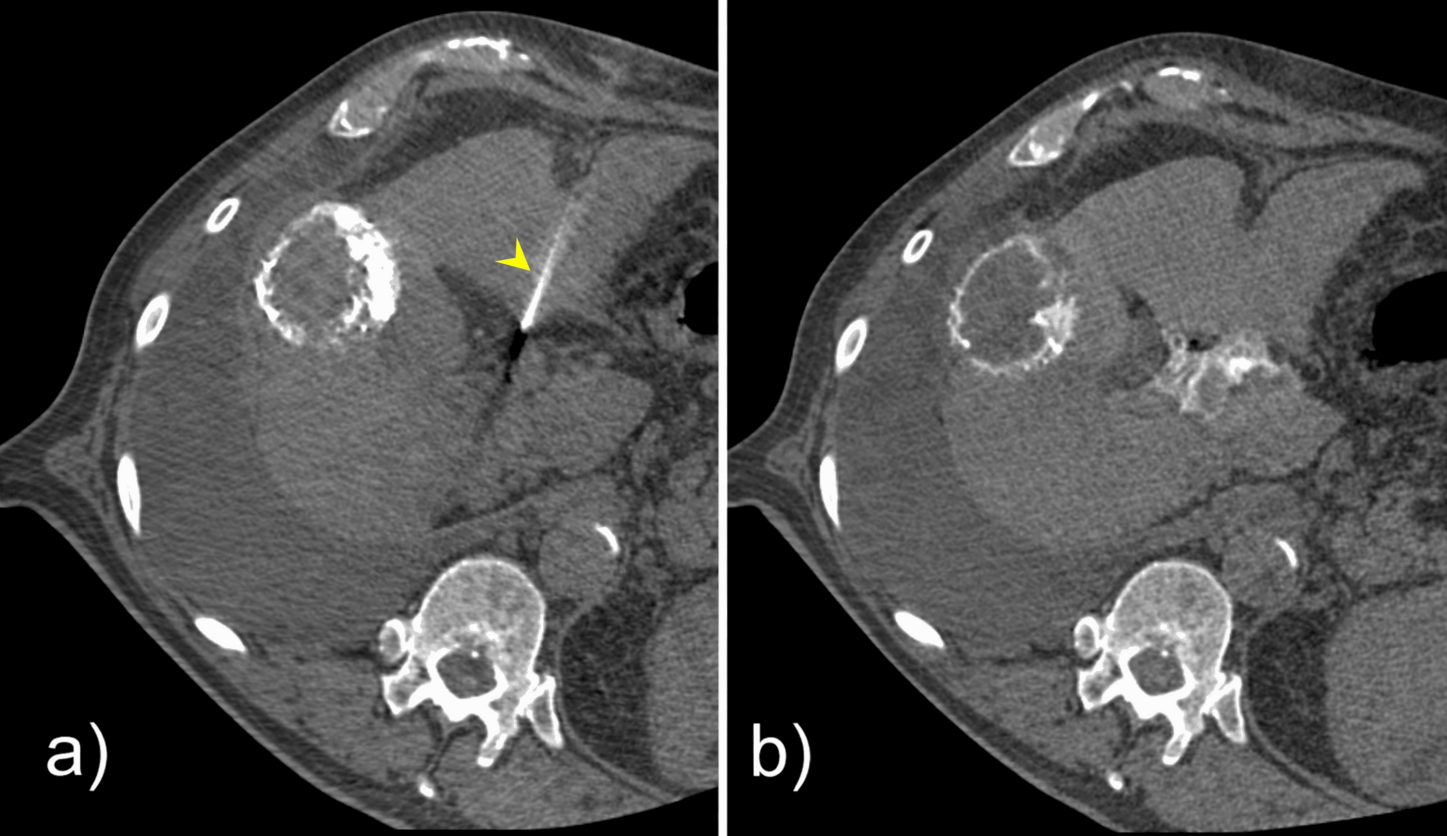
Computed tomography (CT) images of the patient (A) Axial computed tomography (CT) image with 3-mm slice thickness and 1-mm reconstruction interval demonstrating a 22-gauge needle (yellow arrowhead) positioned within the periportal space at the porta hepatis. (B) Repeat axial CT image obtained after administration of 5 mL of iodinated contrast (Visipaque® 320), demonstrating periportal contrast distribution without evidence of intravascular opacification, confirming appropriate needle positioning.

Under computed tomography (CT) guidance with a 3-mm slice thickness and 1-mm reconstruction interval, a 22-gauge, 15-cm needle was advanced into the periportal space at the porta hepatis via a left hepatic lobe approach (Figure 3A). Once appropriately positioned adjacent to the main portal vein, three tests were performed to confirm an extravascular location: (1) aspiration to confirm absence of blood return, (2) injection of 5 mL of iodinated contrast to verify periportal spread along the porta hepatis plexus without intravascular opacification (Figure 3B), and (3) injection of 5 mL of 1% lidocaine with epinephrine under continuous heart rate monitoring to assess for tachycardic response.

Once appropriate positioning was confirmed, a total of 20 mL of 0.5% ropivacaine was injected with intermittent aspiration to ensure the absence of blood return, in accordance with previously described safety protocols [5,8].

Liver lesion MWA

**Figure 4:**
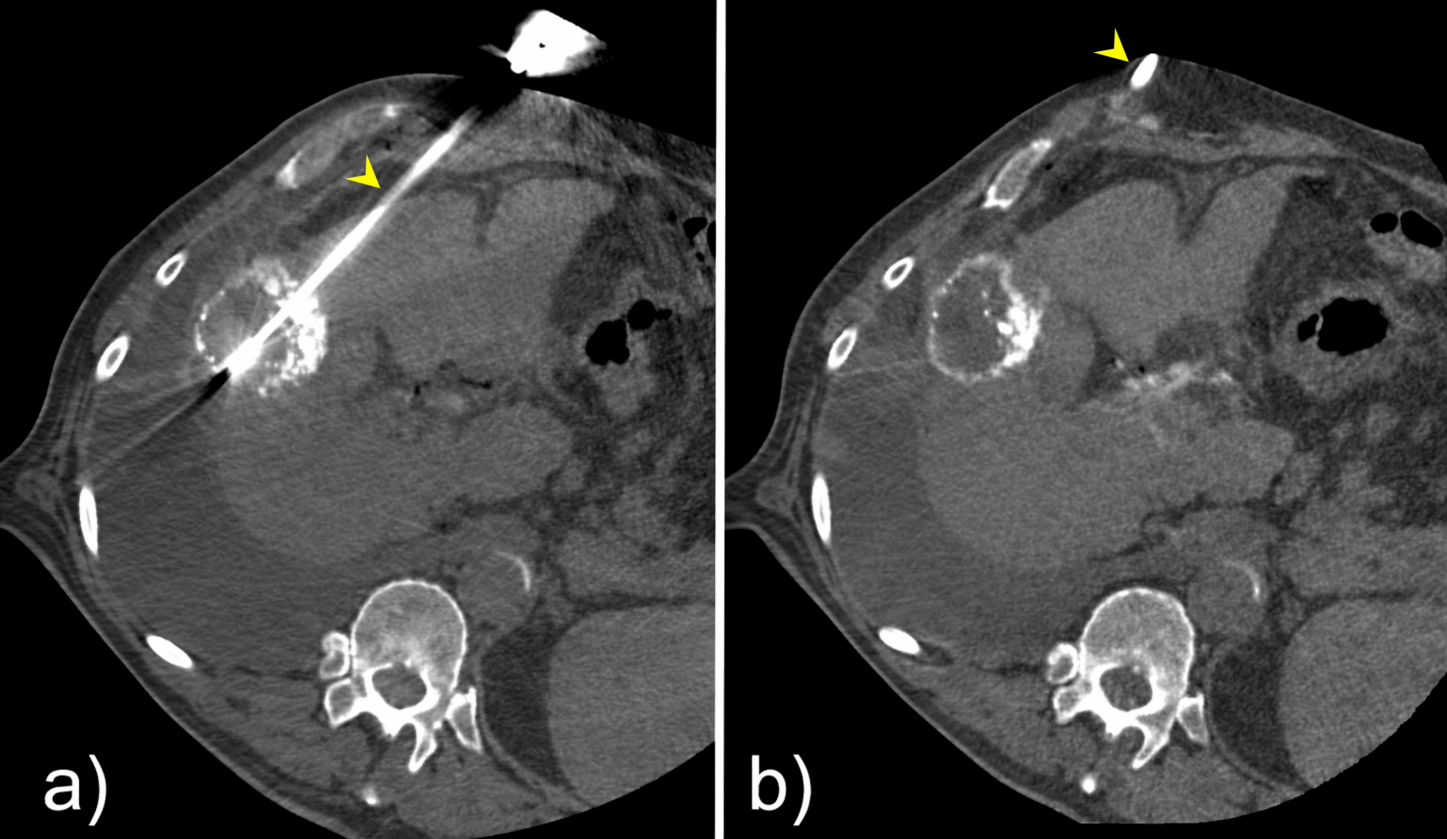
(A–B) Two microwave ablation probes (yellow arrowheads) positioned on the opposite sides of the target lesion within hepatic segment V to ensure adequate ablation coverage

**Figure 5:**
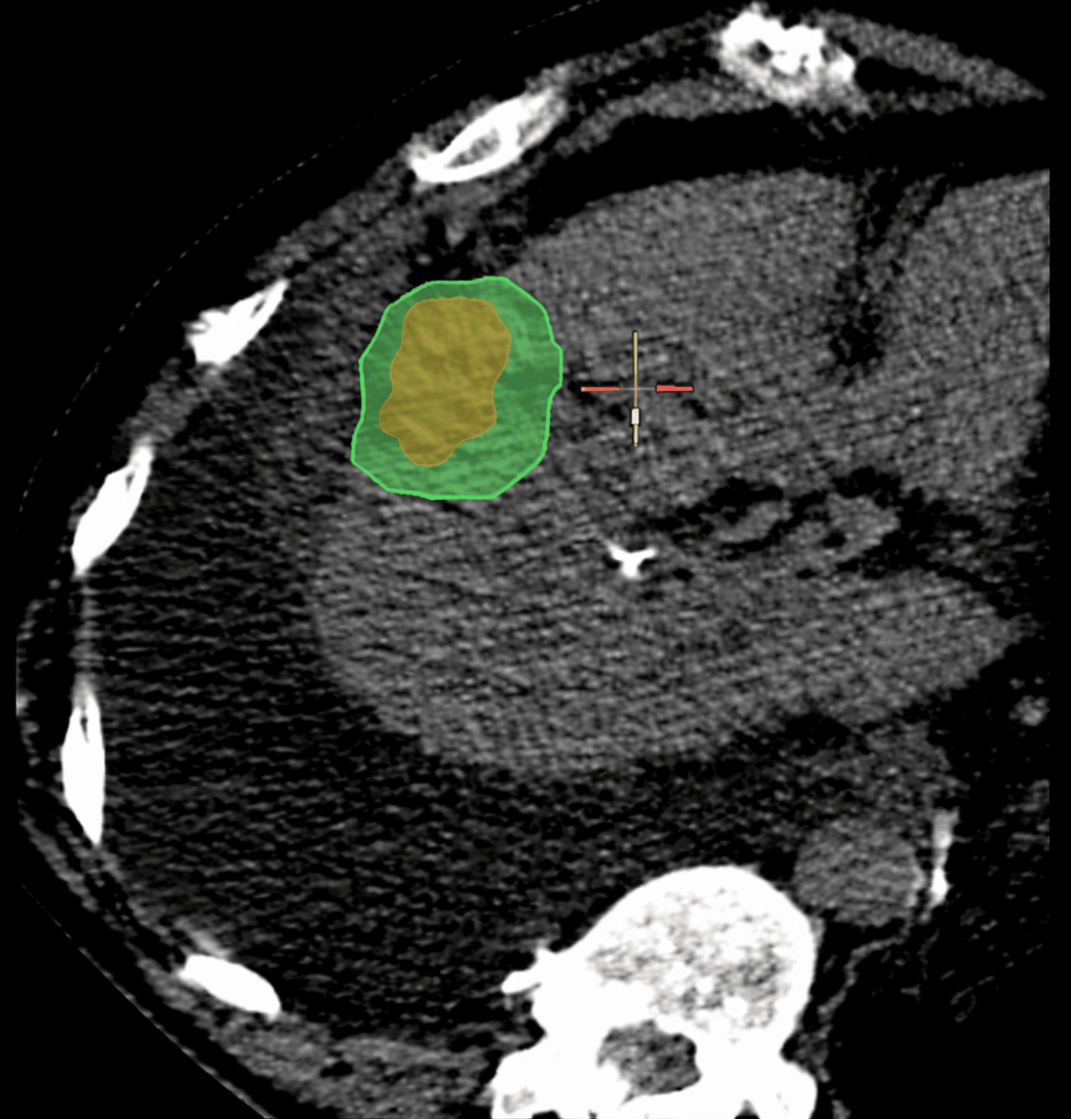
Post-ablation contrast-enhanced computed tomography (CT) demonstrating adequate coverage of the target lesion with appropriate ablation margins, confirmed using image fusion software

Following the successful nerve block, two 15-gauge PRISMA™ MWA probes (NEUWAVE™, Johnson & Johnson MedTech) were positioned within the liver lesion under combined ultrasound and CT guidance (Figure 4). A 10-minute ablation cycle was performed at 70 W, achieving adequate coverage of the target lesion (Figure 5).

The moderate sedation was achieved using 4 mg of midazolam and 200 mcg of fentanyl intravenously, combined with a hepatic hilar nerve block.

The patient remained comfortable and hemodynamically stable throughout the procedure, with no escalation of sedation required, and was discharged on the same day without complications. During hospitalization, the patient reported only mild pain with a visual analog scale (VAS) score of 3/10, controlled with acetaminophen alone, without opioid requirement. At one-week follow-up, the patient reported no pain (VAS 0/10).

## Discussion

Effective pain control is a critical component of percutaneous liver tumor ablation, as inadequate analgesia may compromise patient cooperation, procedural safety, and technical success [4,6]. Visceral hepatic pain is primarily mediated by afferent autonomic fibers innervating the hepatic parenchyma and Glisson's capsule, which are intensely stimulated during high-temperature thermal therapies, such as MWA [4,5]. This visceral pain component is often poorly controlled with conventional moderate sedation alone and may necessitate escalation to deep sedation or general anesthesia [4,9].

The hepatic hilar nerve block specifically targets the hepatic plexus at the porta hepatis, where sympathetic and parasympathetic fibers travel in close proximity to the main portal vein and hepatic artery before entering the liver parenchyma [5,10,11]. Local anesthetic deposition at this strategic location provides direct visceral hepatic analgesia at a proximal point along the pain pathway, offering a mechanistic advantage over regional techniques that primarily address somatic abdominal wall pain [8,11]. By interrupting visceral afferent signaling before hepatic capsular and parenchymal transmission, this approach can significantly improve analgesic efficacy during liver-directed interventions [4,8].

Compared with transversus abdominis plane and paravertebral blocks, which predominantly provide somatic analgesia of the abdominal wall, hepatic hilar nerve block more directly addresses the visceral pain component associated with hepatic interventions [8,11]. In contrast to celiac plexus block, this technique avoids extensive sympathetic blockade and the associated risks of hypotension, diarrhea, and hemodynamic instability in patients with cirrhosis or portal hypertension [12,13]. These characteristics make hepatic hilar nerve block particularly attractive for use in patients with advanced liver disease or significant cardiopulmonary comorbidities and in procedural settings where general anesthesia is undesirable or not readily available [4,8].

The choice of local anesthetics is an important factor in the effectiveness and safety of hepatic hilar nerve block [4]. For example, ropivacaine is well-suited for hepatic hilar nerve block due to its favorable pharmacologic profile, including prolonged duration of action and reduced cardiotoxicity compared with other long-acting amide anesthetics, such as bupivacaine [4,9,11]. Additionally, ropivacaine's lower lipid solubility and relative sensory-selective properties allow for sustained visceral analgesia while minimizing the risk of systemic toxicity, which is important when performing regional blocks in patients with impaired hepatic function [11,14,15]. The extended duration of analgesia provided by ropivacaine may also contribute to improved post-procedural pain control and reduced reliance on opioid medications [4,9].

Beyond percutaneous thermal ablation, the analgesic benefits of ropivacaine-based hepatic hilar nerve block may extend to other liver-directed and hepatobiliary interventions [4,5]. Procedures such as transarterial chemoembolization (TACE), biliary drainage, biliary stenting, and complex hepatic catheter-based therapies are frequently associated with significant visceral discomfort both during and after the intervention [4]. Incorporation of targeted visceral nerve blocks in these settings may enhance patient tolerance, reduce sedation requirements, and improve overall procedural experience [4,5].

Overall, hepatic hilar nerve block using ropivacaine represents a versatile and physiologically targeted analgesic strategy that aligns with the goals of minimally invasive interventional oncology [4,5]. Its ability to provide effective visceral pain control while minimizing anesthetic risk supports its broader consideration as an adjunct to moderate sedation in selected patients undergoing liver-directed procedures [5,8]. Existing retrospective studies confirm that hepatic hilar nerve block significantly reduces required fentanyl and midazolam dosages during ablation [5], with this case report functioning as a technical and educational supplement to the emerging evidence base supporting its use.

## Conclusions

Hepatic hilar nerve block is an effective adjunct for analgesia during percutaneous liver ablation performed under moderate sedation. Current literature suggests that this nerve block technique can improve procedural and post-procedural pain control while reducing postoperative opioid requirements. The hepatic hilar nerve block technique may offer an alternative for patients who are not suitable candidates for general anesthesia and could improve the overall safety and tolerability of percutaneous thermal ablation. However, prospective studies with long-term follow-up are needed to further assess the durability and generalizability of the benefits associated with hepatic hilar nerve block.
